# Editorial: Brain injury in spontaneous intracerebral hemorrhage: from bench to bedside

**DOI:** 10.3389/fstro.2024.1501662

**Published:** 2024-10-30

**Authors:** Zhe Kang Law, Kailash Krishnan, Jatinder S. Minhas, Qi Li

**Affiliations:** ^1^Neurology Unit, Department of Medicine, Faculty of Medicine, National University of Malaysia, Kuala Lumpur, Malaysia; ^2^Stroke, Department of Acute Medicine, Nottingham University Hospitals National Health Service (NHS) Trust, Nottingham, United Kingdom; ^3^Stroke, Academic Unit of Mental Health and Neuroscience, University of Nottingham, Nottingham, United Kingdom; ^4^Cerebral Haemodynamics in Ageing and Stroke Medicine (CHiASM) Group, Department of Cardiovascular Sciences, University of Leicester, Leicester, United Kingdom; ^5^National Institute for Health and Care Research (NIHR) Leicester Biomedical Research Centre, Leicester, United Kingdom; ^6^Department of Neurology, The Second Affiliated Hospital of Anhui Medical University, Hefei, Anhui, China; ^7^Department of Neurology, The First Affiliated Hospital of Chongqing Medical University, Chongqing, China

**Keywords:** intracerebral hemorrhage, brain injury, perihematomal edema, clinical studies, review article, animal studies, blood pressure

Intracerebral hemorrhage (ICH) remains a devastating condition with high mortality and morbidity. Timely administration of a care bundle that includes intensive blood pressure lowering, reversal of anticoagulation, strict glycemic control and treatment of fever significantly improves functional outcome, and reduces mortality (Ma et al., 2023). A subgroup of lobar ICH patients with volume of 30–80 ml and no intraventricular hemorrhage benefits from minimally invasive surgery (Pradilla et al., 2024) whilst decompressive craniectomy may be life-saving (Beck et al., 2024). However, most survivors of ICH remain functionally dependent despite these advances. Understanding the mechanism of brain injury, as well as exploring potential therapeutic targets may help with development of new treatment options. This Research Topic features several studies that span pre-clinical studies exploring mechanism of disease and a potential therapeutic agent as well as clinical studies that explore prognostication of ICH.

A review article on cerebral edema in ICH by Krishnan et al. outlined the complex mechanisms of brain injury after ICH and potential therapeutic targets. Perihematomal edema evolves over three phases: hyperacute, intermediate, and late phases.

The hyperacute phase is characterized by clot retraction, vasogenic, and cytotoxic edema. The activation of SUR1-TRPM4 channels, Na-K-Cl co-transporters, aquaporin channels and increased hydrostatic pressure are involved in the hyperacute phase. The intermediate phase is attributable to activation of thrombin, microglial/macrophage, cytokines, complements, matrix metalloproteinases (MMPs), and breach of blood-brain barrier. The late phase is mediated by toxicity of hemoglobin, iron and reactive oxygen species as a result of erythrolysis.

The authors also reviewed clinical trials that targeted cerebral edema, including osmotic agents, antihypertensives, neurosurgery, anti-inflammatory agents, and agents targeting erythrocyte degradation products but conclude that there is currently no established treatment to reduce or prevent perihematomal edema. Amongst other things, the authors suggest that agents targeting neuroinflammation, free radicals and neuroprotection may be promising. Combining anti-edema treatment with other established acute treatments such as blood pressure lowering or reversal of coagulopathy may be considered when designing future clinical trials.

A potential neuroprotectant is explored in a mouse study by Wu Y. et al. Pterostilbene (3′, 5′-dimethoxy-resveratrol) is a *trans*-stilbene compound found in the herb red sandalwood, which has a variety of putative properties such as antioxidant, anti-tumor, hypolipidemic and bacteriostatic. In this study, Pterostilbene reduced ICH volume and neural apoptosis, and alleviated blood-brain barrier (BBB) damage and cerebral edema, with improvement in neurological behavior tests. Pterostilbene acted as neuroprotectant by suppressing microglia-derived inflammation in mice through OPA1 mediated remodeling of mitochondrial dynamics.

A better understanding of the pathophysiology of ICH is important to identify therapeutic targets and this is studied in two clinical studies. Yu et al. explored the role of matrix metalloproteinase-2 (MMP-2) in ICH. In an observational study, the authors measured MMP-2 levels in 93 ICH patients within 24 h of symptoms onset and 88 healthy controls and found that MMP-2 levels were reduced in ICH patients compared to healthy controls. Lower MMP-2 levels were associated with greater perihematomal edema volume and worse clinical outcome (higher NIHSS and lower Glasgow Coma Scale, GCS). Multivariable regression analysis adjusting for key prognostic factors including hematoma volume showed that lower MMP-2 levels were independently predictive of greater edema volume. It is postulated that leukocyte and complement C4 lactivation lead to formation of membrane-attack complex (MAC) which form pores on membranes of glia, neurons, and endothelial cells. These pores inhibited the formation of a MMP-2, TIMP-2, and MMP-14 trimolecular complex in the cellular membrane. Therefore, a lower MMP-2 and MMP-14 levels is indicative of a inflammatory response.

Moullaali et al. explored the role of blood pressure variability in different sub-types of ICH. This was a prospective, population-based, inception cohort study which explored the association between systolic blood pressure variability (SBPV) during 10 years before first-ever ICH onset in adults who died with presence and severity of cerebral amyloid angiopathy (CAA) on autopsy. The study included 72 patients (34 patients with moderate to severe CAA and 38 patients with mild or absent CAA) with 62 patients had at least 2 BP measurement for primary measure of SBPV. Lower maximum and range of SBP were significantly associated with moderate-severe CAA after adjustment for mean SBP on binary logistic regression analysis. These findings support that the role of blood pressure variability in the pathogenesis of the two main cerebral small-vessel disease sub-types that cause ICH is different, where the role is likely more critical in hypertensive ICH, whilst less significant in CAA-ICH.

Prognostication of ICH is important as well to identify patients at risk of adverse outcome. Feng et al. performed a single-center retrospective study of 269 patients aiming to construct a prediction model for the prognostication of ICH, with a focus on cerebral microbleeds (CMB). The outcomes of interest were poor functional outcome (modified Rankin Scale, mRS > 2) and mortality at discharge, 3 and 12 months. The authors found that GCS, National Institutes of Health Stroke Scale (NIHSS) and hematoma volume predicted mRS at discharge; GCS, NIHSS, epencephalon hemorrhage and hematoma volume predicted 3-month mRS whilst GCS, NIHSS, and neurosurgery predicted 12-month mRS. Hematoma volume was the only independent predictor of 12-month mortality. All predictive models have a good AUROC of ~0.87. Notably, cerebral microbleeds, whether deep or lobar, did not predict of functional outcome or death. Although CMB is a known risk factor for recurrent ICH and ischemic stroke, this was not assessed by the authors.

In conclusion, the collection of articles in this Research Topic provides an insight into the mechanisms of brain injury in ICH as well as specific therapeutic targets. This editorial highlights progress and innovations that have been made including data that are emerging to enable translation from pre-clinical to risk stratification of patients and in ongoing randomized trials. ICH is a medical emergency, warrants clinical prioritization and this editorial highlights clearly that this high-risk patient population warrant greater research focus. Ultimately, the best treatment of ICH is prevention and effective detection. Control of hypertension may have the greatest effect on reducing the global burden of ICH. Continuing pre-clinical coupled with translational clinical research is needed to further advance treatment in ICH, hence the theme of this Research Topic: *from bench to bedside*.
